# Internal Fixation of Garden Type III Femoral Neck Fractures with Sliding Hip Screw and Anti-Rotation Screw: Does Increased Valgus Improve Healing?

**DOI:** 10.3390/medicina58111573

**Published:** 2022-11-01

**Authors:** Simon Hackl, Christian von Rüden, Ferdinand Weisemann, Isabella Klöpfer-Krämer, Fabian M. Stuby, Florian Högel

**Affiliations:** 1 Department of Trauma Surgery, BG Unfallklinik Murnau, 82418 Murnau, Germany; 2 Institute for Biomechanics, Paracelsus Medical University, 5020 Salzburg, Austria; 3 Institute for Biomechanics, BG Unfallklinik Murnau, 82418 Murnau, Germany

**Keywords:** femoral neck fracture, Garden classification, sliding hip screw, anti-rotation screw, valgus reduction, Kellgren–Lawrence score, outcome

## Abstract

*Background and Objectives:* The aim of this study was to compare the effect of valgus versus anatomic reduction on internal fixation of Garden type III femoral neck fractures using the sliding hip screw (SHS) and anti-rotation screw (ARS) regarding the radiographic and therapeutic outcome. *Patients and Methods:* A retrospective case-controlled study was performed in a level I trauma center. All patients between 2006 and 2020 aged younger than 70 years with a Garden type III femoral neck fracture and a Kellgren–Lawrence score under grade III stabilized using SHS and ARS were identified. One-hundred and nine patients were included, with a group distribution of sixty-eight patients in group A (anatomic reduction) and forty-one patients in group B (valgus reduction). *Results:* Mean age was 55 years, and the mean Kellgren–Lawrence score was 1 in both groups. Mean femoral neck angle was 130.5 ± 3.8° in group A and 142.8 ± 4.3° in group B (*p* = 0.001), with an over-correction of 12° in group B. Tip-apex distance was 10.0 ± 2.8 mm in group A versus 9.3 ± 2.8 mm in group B (*p* = 0.89). Healing time was 9 weeks in group A compared to 12 weeks in group B (*p* = 0.001). Failure rate was 4.4% in group A and 17.1% in group B (*p* = 0.027). *Conclusions:* Anatomic reduction of Garden type III femoral neck fractures in patients younger than 70 years treated using SHS and ARS resulted in significantly lower failure rates and shorter healing times than after valgus reduction. Therefore, it can be recommended to achieve anatomic reduction.

## 1. Introduction

Younger adults are more likely to suffer from unstable Garden type III femoral neck fractures demanding accurate reduction and stable internal fixation, while having a higher likelihood of failure due to missing intrinsic instability [1,2,3]. Although there is still a lack of consensus regarding the most appropriate fixation technique for femoral neck fractures, sliding hip screw (SHS) supplemented with a cannulated anti-rotation screw (ARS) is commonly accepted as one of the gold standards for vertical fractures of the femoral neck in younger patients [4]. Apart from the anatomic reduction of these fracture configurations and the optimal restoration of the femoral neck-shaft angle (caput-collum-diaphyseal angle, CCD), the desirable or undesirable possibility of a valgus reduction occurs. In addition to total hip arthroplasty, in cases of failed fixation of femoral neck fractures, an often-described salvage procedure is valgus intertrochanteric osteotomy to achieve valgus positioning of the proximal fracture fragment [5]. Hereby, a larger CCD could be achieved, which converts shear forces at the femoral neck to compressive forces to improve osseous healing, resulting in suitable outcomes and adequate healing rates [5,6,7,8]. Consequently, a potential approach to further minimize complications and to maximize healing rates even in the initial surgical treatment of displaced femoral neck fractures might be to perform the fracture reduction in a slight valgus position of the proximal fragment prior to fracture fixation. Therefore, the aim of this study was to compare the effect of valgus alignment with the effect of anatomical reduction during closed internal fixation of Garden type III femoral neck fractures regarding the radiographic and therapeutic outcome, as well as the time period until osseous healing, using SHS combined with ARS. It was hypothesized that there would be faster and improved fracture healing and a better clinical outcome following valgus reduction of the fracture than after anatomic fracture reduction.

## 2. Patients and Methods

### 2.1. Study Design

**Figure 1:**
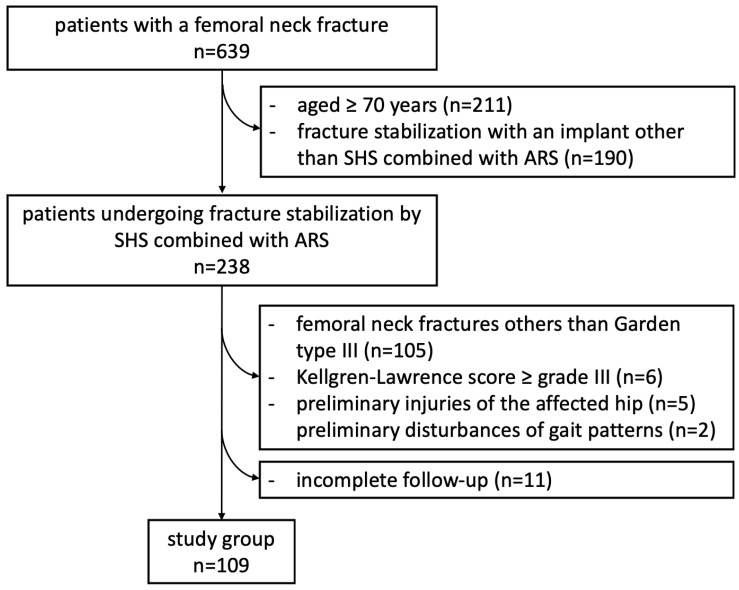
Overview on patients’ inclusion process.

A retrospective case-controlled study was performed in a single European level I trauma center. All patients between 2006 and 2020 aged younger than 70 years suffering a femoral neck fracture diagnosed by biplanar conventional radiographs and stabilized by using SHS combined with ARS were identified. Whenever conventional radiographs were not conclusive to determine the diagnosis or the classification of a femoral neck fracture, computed-tomography (CT) scan was performed to clarify the type of fracture. Garden classification was used to identify the fracture pattern [9]. To minimize inter-observer variation, all fractures were assessed by two experienced senior surgeons and only fractures that were identically classified by both surgeons were included [10]. To objectify the severity of the injury and the morbidity of the study group only patients with a Garden type III femoral neck fracture and a Kellgren-Lawrence score under grade III were included in the study after analyzing the radiological images taken on the day of accident [11,12,13]. Patients with preliminary disturbances of gait patterns and injuries of the affected hip as well as patients with an incomplete follow-up were excluded. After checking the mentioned inclusion and exclusion criteria, the data sets of 109 patients (age 55 ± 11 years; 63 males, 46 females) were included in the analysis (Figure 1).

### 2.2. Surgical Procedure

All surgical procedures were performed in a standard manner under the supervision of twelve experienced senior surgeons. Patients were positioned on the extension table and closed reduction was performed either in anatomic or valgus position as decided by the surgeon—preoperatively and independently of individual patient and fracture criteria—with biplanar X-ray control. Following preparation to the proximal femur region and correct positioning of the aiming device for SHS, a 2.5 mm guide wire was placed center–center into the femoral head to the subchondral area. The position in the femoral neck was aimed to be in the caudal-dorsal quarter. After positioning of the guide wire and biplanar fluoroscopic control, a second 3.2 mm guide wire for the ARS was placed parallel to the first wire, cranially. After measuring the length of the SHS (DHS System, Synthes GmbH, Oberdorf, Switzerland), the femoral neck was prepared using the three-step drill, which was adjusted 10 mm shorter than the measured length following the insertion of the SHS over the guide wire. This procedure was followed by measuring the length of the ARS and insertion by using a cannulated 6.5 mm partial threaded screw (Asnis™ III, Stryker Trauma AG, Selzach, Switzerland), reaching the subchondral area, too. After removing the guide wires and after another X-ray control of the correct positioning of the SHS and ARS, the 2- or 4-hole SHS plate with a barrel angle of 130° or 135° (DHS System, Synthes GmbH, Oberdorf, Switzerland) was fixed to the SHS and the proximal femoral shaft with 2 or 4 bi-cortical 4.5 mm cortex screws. After surgical stabilization of the femoral neck, fracture pain-adapted full-weight-bearing was allowed for all patients.

### 2.3. Clinical and Radiological Assessment

**Figure 2:**
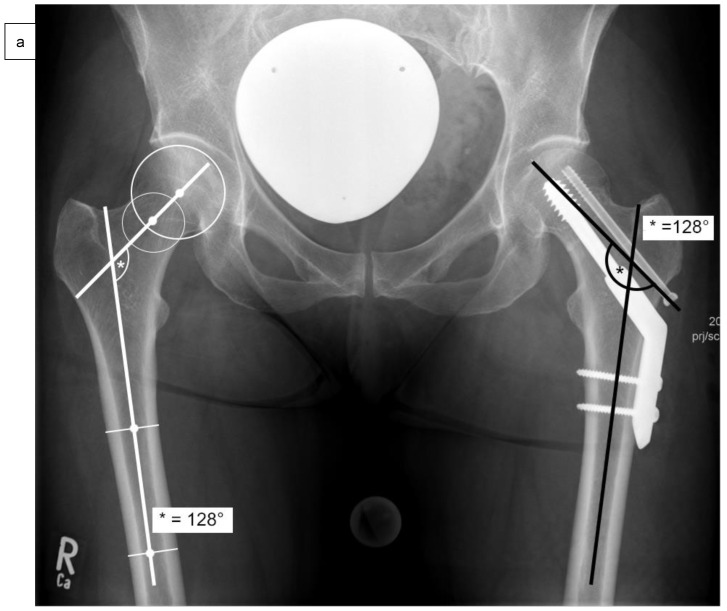

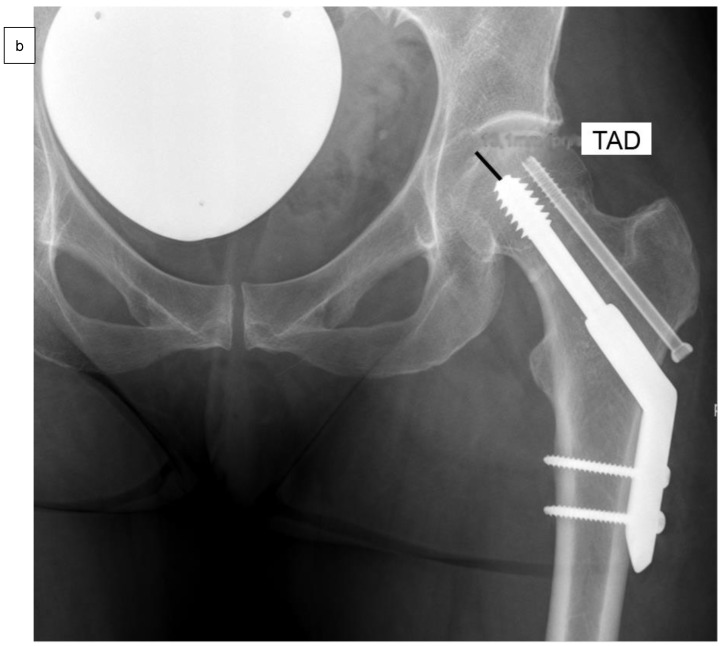

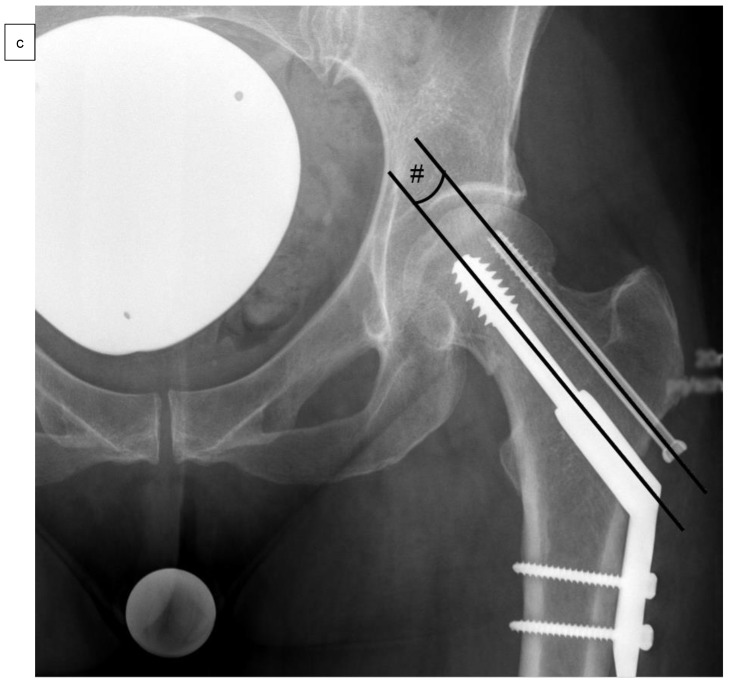

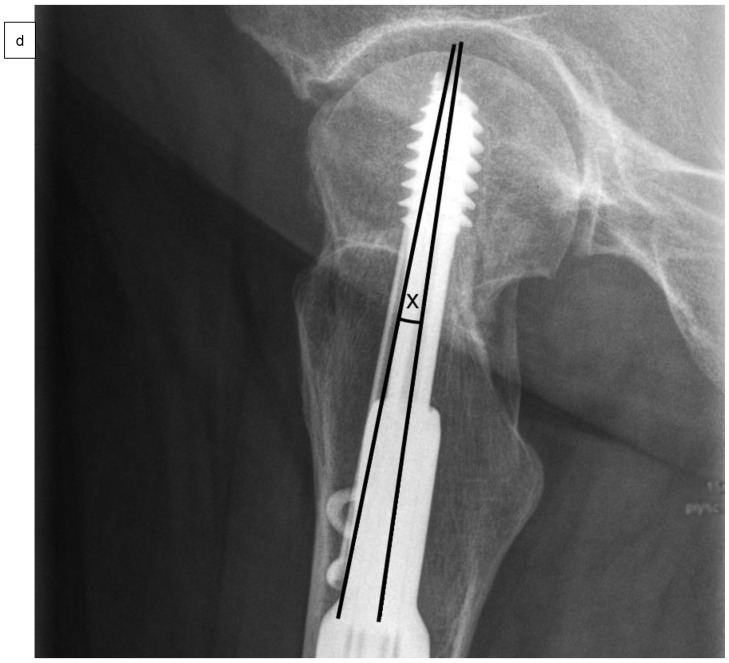
Measurement of the femoral neck angle (*) compared to the contralateral side (°), defined as the angle between the femoral neck axis and the bisecting line of the femoral shaft (**a**). The tip-apex distance (TAD) was defined as the calibrated summation of the distance between the tip of the SHS and the apex of the femoral head on anteroposterior and (not demonstrated) lateral radiographs (mm) (**b**), and the angle between SHS and ASR in the frontal plane (#) (**c**) and the axial plane (x) (**d**) by using biplanar conventional radiographs, six weeks postoperatively.

As well as epidemiological patient parameters and the Kellgren–Lawrence score at the time of hospital admission, the femoral neck angles (°) were captured in comparison to the opposite side (Figure 2a) after reduction and surgical stabilization six weeks postoperatively by using biplanar conventional radiographs. Valgus reduction was defined as a femoral neck angle of more than 5° in comparison to the opposite side. Then, the cohort group was divided into group “A”, which included patients with anatomical reduction, and group “B”, which consisted of patients with valgus reduction of the Garden type III femoral neck fracture. Further on, the angle of the 2- or 4-hole SHS plate (°), the tip-apex distance (mm) as described by Baumgaertner et al. (Figure 2b), and the angle between SHS and ARS (°) were measured in frontal (Figure 2c) and axial planes (Figure 2d) of the intraoperative or postoperative X-rays [14].

In each follow-up visit, the healing time (weeks) and failure rate as well as potential surgery-related complications were examined. Hereby, treatment failure was defined as cutting out of the SHS, and respectively the ARS, collapse of the femoral head, implant loosening, or failure of fracture healing up to 6 months after surgical stabilization. Fracture healing was defined as osseous union of at least three cortices diagnosed by biplanar conventional radiographs [15].

### 2.4. Follow-Up

After discharge from the hospital, patients were followed-up clinically and radiologically in the outpatient department at regular intervals after 6 weeks, followed by 4-week intervals until the sixth month after surgical stabilization of the femoral neck fracture or until fracture healing was documented. In addition, in case of suspected complications, additional visits were scheduled. During each visit, patients where clinically and radiologically examined regarding fracture healing and possible complications. Furthermore, in addition to these documented follow-up examinations, the conventional radiographs were retrospectively re-evaluated by two experienced senior surgeons to verify the healing time, defined as the time from the diagnosis of the femoral neck fracture to its osseous consolidation.

### 2.5. Statistical Analysis

As well as implant-related parameters, tip-apex distance, Kellgren–Lawrence score, healing time and treatment failure were compared between groups A and B (SPSS version 26.0, SPSS Inc., Chicago, IL, USA). For all variables, a check for normal distribution was performed using the Kolmogorov–Smirnov test. Only two variables from the group of valgus patients (group B) demonstrated a normal distribution, so the non-parametric Mann–Whitney test was used. Due to multiple testing, for the comparison of the three variables healing time, femoral neck angle and tip-apex distance, the significance level was set to α = 0.05/3 = 0.017. The complications (dichotomous yes/no) were tested for a significant difference between the groups using Pearson’s chi-square test (α = 0.05). Effect sizes for significant differences were calculated using Cohen’s d (for the Mann–Whitney test) and Phi’s r (for Pearson’s chi-square test) [16]. Results of this study are presented as mean values ± standard deviation. Results were considered statistically significant with *p* values < 0.05.

### 2.6. Ethics and Study Registration

The study adhered to the tenets of the Helsinki Declaration and according to the guidelines and the approval of the Ethics Committee of the institutional and national Medical Board (Bavarian State Chamber of Physicians, ID 2022-1157). On 2 August 2022, the study was retrospectively registered with the German Clinical Trials Register (Trial registration number: DRKS00029953).

## 3. Results

### 3.1. Patient Cohort

One-hundred and nine patients were included in the study, with a group distribution of sixty-eight patients in group A with anatomical and forty-one patients in group B with valgus reduction of the Garden type III femoral neck fracture (Table 1).

The average femoral neck angle was significantly different between groups A and B (group A: 130.5 ± 3.8°, group B: 142.8 ± 4.3°; *p* = 0.001, effect size d = 0.829). In accordance with the inclusion criteria, the over-correction in comparison to the opposite side was performed with 12 ± 4.2° in group B and 1.2 ± 1.3° in group A (*p* = 0.001). Average age was 55 years in both groups (group A: 55 ± 11 years, group B: 55 ± 11 years). In addition, with an average Kellgren–Lawrence score of 1 in both groups, no significant difference could be observed (group A: 1.0 ± 0.6, group B: 1.2 ± 0.6). Regarding the tip-apex distance with 10.0 ± 2.9 mm in group A versus 9.3 ± 2.8 mm in group B (*p* = 0.89), no significant difference could be detected. In the axial plane of the biplanar X-rays, the mean angle between SHS and ARS in group A was 2.2 ± 1.8°, and 2.4 ± 1.5° in group B (*p* = 0.76). In the frontal plane of the biplanar X-rays, the screws were almost exactly parallel in both groups (group A: 0.0 ± 0.2°, group B: 0.0 ± 0.3°; *p* = 0.56). Considering the duration of the surgical procedure—defined by cut-to-seam time—as a parameter for its difficulty, no significant difference could be observed in both groups (group A: 58 ± 19 min, group B: 60 ± 21 min; *p =* 0.70).

### 3.2. Treatment Failure

**Figure 3:**
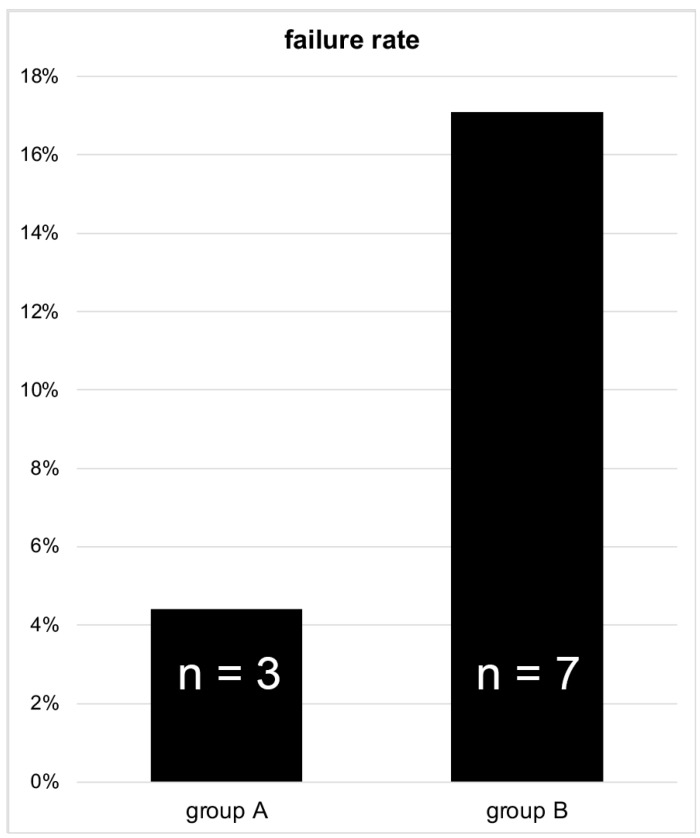
Comparison of the failure rate in surgically treated Garden type III femoral neck fractures.

Regarding the failure rate of the surgical-stabilized femoral neck fractures, a relevant difference could be observed between patients who received an anatomical and those who received a valgus reposition. The failure rate was significantly higher in group B than in group A, as 7 complications (17.1%) appeared after valgus reduction in group B and 3 cases with fracture-related complications (4.4%) were detected after anatomical reduction in group A (*p* = 0.027, effect size r = 0.212) (Figure 3).

In detail, in group A, two cases of femoral head necrosis were observed, while one of these cases was stabilized more than 24 h after trauma and one cutting out of the SHS was found. In group B, femoral head necrosis was found in five cases and cutting out of the SHS in two cases. All these patients had to be revised by total hip arthroplasty (THA) due to immobilizing hip pain. Implant loosening or failure of the fracture to heal over 6 months were not observed in any patient.

### 3.3. Healing Time

**Figure 4:**
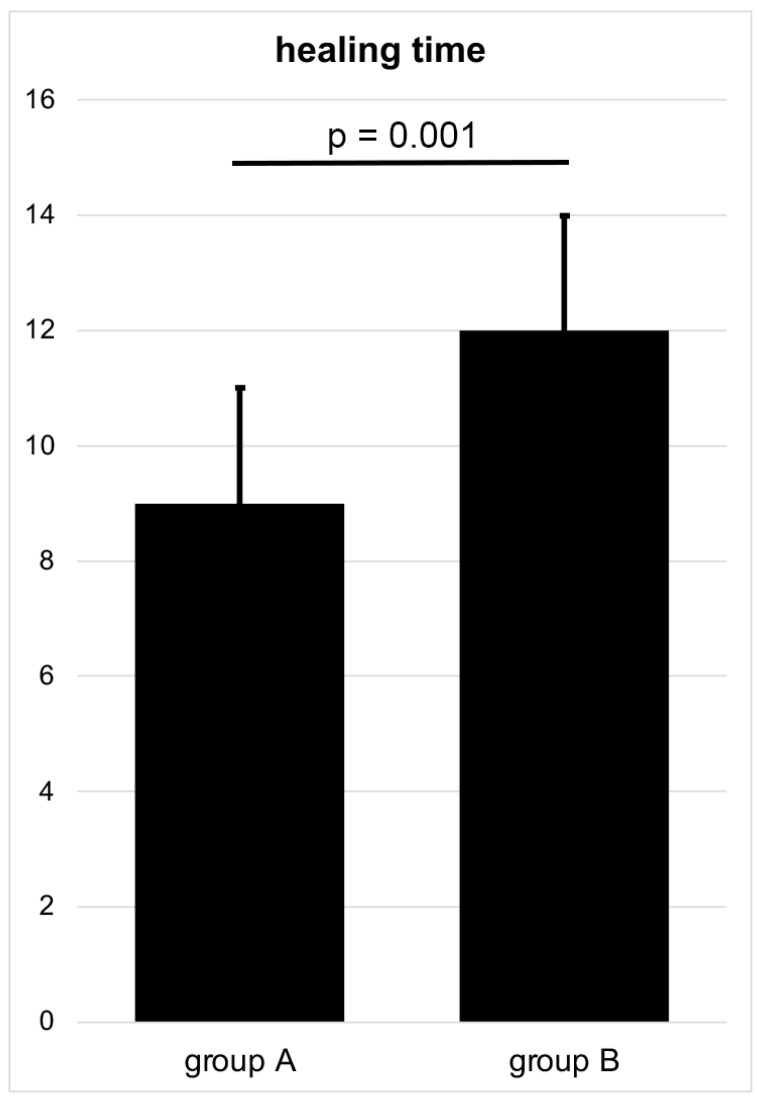
Duration of time to osseous healing after anatomical and valgus reduction stabilized using SHS and ARS (weeks) in a Garden type III femoral neck fracture.

In accordance with the above-mentioned treatment failure, osseous healing of the Garden type III femoral neck fracture stabilized by SHS and ARS could be achieved in group A in 65 out of 68 patients and in group B in 34 out of 41 patients during the follow-up period of 6 months, accompanied by full-weight-bearing. Hereby, osseous healing was significantly shorter after anatomical reduction in group A, with a mean healing time of 9 ± 2 weeks compared to 12 ± 2 weeks after valgus reposition in group B (*p* = 0.001, effect size d = 0.509) (Figure 4).

## 4. Discussion

The objective of this study was to evaluate the potential influence of valgus reduction on the healing process in Garden type III femoral neck fractures fixed using SHS in combination with ARS. Despite the promising outcome of valgus intertrochanteric osteotomy as well as biomechanical considerations, performing the fracture reduction in Garden type III femoral neck fractures in a slight valgus position of the proximal fragment prior to fracture fixation with SHS and ARS was not superior compared with the anatomical reduction in patients younger than 70 years. In the current study, an average valgus correction of femoral neck fractures of 12° resulted in a significantly increased rate of treatment failure as well as in a longer period of time until fracture healing of about 3 months. In so far, based on the results of this study in patients younger than 70 years, anatomical reduction of Garden type III femoral neck fractures is suggested prior to internal fixation with SHS and ARS.

There are several studies examining the outcome and the biomechanical behavior after femoral neck fractures regarding the different types of fracture fixation, demonstrating an advantage of the sliding hip screw systems in combination with an additional anti-rotation screw [1,17]. Nevertheless, studies regarding the influence of the reduction of the proximal fragment of femoral neck fractures are rare and difficult to compare due to the different methods of fracture stabilization. Regardless of this, femoral neck fractures fixed in a varus position demonstrated the highest rate of failure (37.5%), followed by fractures fixed with a visible medial transcervical line independent of anatomical or valgus reduction of the proximal fragment (36.0%) [18]. In a recently published finite element analysis, anatomic reduction of valgus-impacted femoral neck fractures diminished the stress at the fracture ends, although displacement significantly increased. When the fracture was fixed with SHS and ARS, there was less stress at the fracture end with anatomic reduction than without [19]. For example, Ramallo et al. examined 81 patients younger than 60 years with femoral neck fractures treated with closed reduction and internal fixation using three cannulated screws and evaluated a significant influence of a satisfactory reduction on the outcome [20]. Satisfactory reduction was defined as deviation of the focus of the fracture less than 2 mm in combination with Garden angles between 160° and 180°. After sufficient reduction, treatment failure was observed in 7% compared with an over 8 times higher risk of failure when the reduction was inadequate. Although the failure rate of satisfactory reductions was comparable to our failure rate of 4% after anatomic reduction, no statement was made regarding valgus positioning by Ramallo et al. [20]. Schwartsmann et al. detected a failure rate in femoral neck fractures after good reduction—defined by a normal or slightly valgus reduction in accordance with the alignment index stated by Garden—and stabilization with a sliding hip screw system in about 20% of the cases, comparable to our failure rate of 17.5% after valgus reduction [21,22]. Incorrect screw positioning in the femoral head was identified as a main risk factor for therapy failure due to necrosis [21]. A comparable failure rate of 19.5% to the valgus group of the current study was observed after fixation of femoral neck fractures with valgus impaction and any further reduction [23]. Although there are studies which did not find any affection of the healing rate by the quality of reduction [24,25], failure rates in the present study correlate with the majority of the current literature, highlighting the need of an appropriate reduction of femoral neck fractures. A closer look at the type of complications showed that in total, 7 out of 109 patients analyzed (6.4%) developed femoral head necrosis. In particular, after anatomic reduction, femoral head necrosis occurred in only 2 of 68 patients (2.9%) and after valgus reduction in 5 of 41 patients (12.2%) during follow-up. Considering the frequency of avascular necrosis of the femoral head after stabilization of Garden type III femoral neck fractures in the current literature, with a 95% confidence interval between 6.4% and 27.6%, the incidence of femoral head necrosis in our study cohort is quite low, especially after anatomic reduction [26]. One possible reason for this moderate necrosis rate could be—as noted in a recent meta-analysis—that femoral neck systems present a lower rate of femoral head necrosis than cannulated cancellous screws, which are also commonly used worldwide and thus may have an impact on the previously observed necrosis rates [27].

A more detailed look at the group of patients with an acceptable reduction of the femoral neck fracture demonstrated a mixed picture in the available literature: A study published in 1981 after evaluation of 446 cases of femoral neck fractures in all Garden’s stages treated by internal fixation reported a healing rate of 90% after a reduction in valgus position and only 77% if anatomical reduction was achieved [28]. In addition, Füchtmeier et al. analyzed a patient cohort of 51 patients during 1975 and 1985 and reported that the anatomical reduction of femoral neck fractures—compared to a valgus position—and stabilization with mainly three to four cannulated screws resulted in a higher, but statistically not significant, rate of osteonecrosis (18.2%) and nonunion (9.1%) in the first 5 years following osteosynthesis [29]. These findings are in contrast to the present study, demonstrating a lower rate of treatment failure after anatomical reduction, with 4%, which is more interesting since in both studies, the average valgus correction of femoral neck fractures was about 12°. One possible explanation for these opposite findings might be the utilized stabilization with SHS and ARS instead of mainly cannulated screws, as well as that the patients’ cohort includes all Garden’s stages with a significantly higher rate of Garden type III femoral neck fractures in the group with valgus position. Looking at the group of patients with anatomical reduction between 5 and 15 years after internal fixation analyzed by Füchtmeier et al., a change in both groups was observed, and a better long-term outcome with an improved functional outcome and a lower rate of osteoarthritis of the hip were detected in the group that received anatomic reduction [29]. Focusing on therapy failure by analyzing 202 patients with femoral neck fractures treated using 3 cannulated screws, Yang et al. did not find any significant difference regarding development of nonunion after anatomical reduction [30]. Looking at the failure rate in relation to the size of the valgus tilt of valgus-impacted femoral neck fractures which were not dis-impacted and were stabilized with three cannulated screws, Song et al. could demonstrate that a valgus tilt above 15° results in a higher failure rate [31].

Another important point that supports the anatomical reduction of femoral neck fractures in addition to the above-mentioned lower risk of developing osteoarthritis of the hip in younger patients is the higher rate of femoral neck shortening in valgus-impacted femoral neck fracture combined with a worse functional outcome due to a femoral offset shortening and the resulting change in the abductor moment arm [31]. This is also emphasized by a study conducted by Park et al., which demonstrated that anatomical reduction of femoral neck fractures with valgus impaction above 15° leads to a better functional outcome one and two years following osteosynthesis, compared to in situ stabilization of the valgus-impacted femoral neck fractures without any reduction [25].

Relevant literature data regarding the time period between surgery and fracture healing could not be found yet. The results of the present study demonstrated a significantly shorter healing time after anatomic reduction of the femoral neck, which may be explained by less compromise of blood supply than after valgus reduction of the femoral neck.

Limitations of this study might be seen in the retrospective study design, resulting in a lack of clarity as to why in every single case, the surgical team decided for anatomical or valgus reduction. Besides, a longer follow-up for at least one year would be helpful to investigate if early secondary THA would have been needed due to the offset change related to the femoral neck angle reduction. Nevertheless, to our knowledge, the current study is the first clinical trial comparing SHS in combination with additive ARS in an excellent comparable patients’ cohort consisting only of Garden type III femoral neck fracture stabilized by the same implants, with a focus on the intraoperative reduction angle of the femoral neck.

## 5. Conclusions

Anatomic reduction of Garden type III femoral neck fractures in patients younger than 70 years treated using SHS in combination with ARS resulted in significantly lower failure rates and shorter healing times than after valgus reduction between 5° and 15° of the proximal fracture fragment. Therefore, based on the results of the current study, it can be recommended to aim for anatomic reduction when using SHS and ARS for internal stabilization.

## Figures and Tables

**Table 1:** Overview of the patient cohort with Garden type III femoral neck fractures divided into group A (anatomic reduction) and group B (valgus reduction). Values are presented as mean ± standard deviation or as total number of patients.

	Group A	Group B	*p*-Value
**Group size** Male Female	68 40 28	41 23 18	
**Age**	55 ± 11 years	55 ± 11 years	0.78
**Body mass index**	24.9 ± 3.2 kg/m^2^	23.6 ± 3.5 kg/m^2^	0.07
**ASA ^1^**	1.6 ± 0.7	1.8 ± 0.6	0.26
**Duration between trauma and fracture stabilization**			
≤24 h >24 h	65 3	40 1	
**SHS plate**			
2-hole 4-hole	61 7	38 3	
**Barrel angle of SHS**			
130° 135°	7 61	3 38	
**Kellgren–Lawrence score**	1.0 ± 0.6	1.2 ± 0.6	0.14
**Cut-to-seam time of the surgical procedure**	58 ± 19 min	60 ± 21 min	0.70
**Tip-apex distance (TAD)**	10.0 ± 2.9 mm	9.3 ± 2.8 mm	0.89
**Angle between SHS and ARS in frontal plane**	0.0 ± 0.2°	0.0 ± 0.3°	0.56
**Angle between SHS and ARS in axial plane**	2.2 ± 1.8°	2.4 ± 1.5°	0.76
**Femoral neck angle 6 weeks postoperatively**	130.5 ± 3.8°	142.8 ± 4.3°	0.001
**Difference of the femoral neck angle 6 weeks postoperatively in comparison to the contralateral side**	1.2 ± 1.3°	12.0 ± 4.2°	0.001

^1^ American Society of Anesthesiologists physical status classification.

## Data Availability

The data presented in this study are available on request from the corresponding author. The data are not publicly available due to privacy.
